# Passive Inclination Sensor Based on a Patch Antenna with a Reconfigurable Water Load

**DOI:** 10.3390/s24206744

**Published:** 2024-10-20

**Authors:** Zhuoran Yi, Zihan Xia, Xianzhi Li, Kangqian Xu, Liyu Xie, Songtao Xue, Yiyu Wu

**Affiliations:** 1 Department of Disaster Mitigation for Structures, Tongji University, Shanghai 200092, China; linsmyk@gmail.com (Z.Y.); xzhooo@tongji.edu.cn (Z.X.); liyuxie@tongji.edu.cn (L.X.); xue@tongji.edu.cn (S.X.); 2 School of Civil Engineering, Qingdao University of Technology, Qingdao 266520, China; lixianzhi@qut.edu.cn (X.L.); xukangqian0826@163.com (K.X.); 3 Department of Architecture, Tohoku Institute of Technology, Sendai 982-8577, Japan; 4 College of Civil Engineering and Architecture, Jiaxing University, Jiaxing 314001, China

**Keywords:** historical building, health monitoring, inclination detection, patch antenna, passive, water load

## Abstract

In order to ensure the safety and preserve the value of historical buildings, inclination is an essential parameter during the continuous structural health monitoring process. However, the wire and price of a traditional sensor limit application. This paper proposes a low-cost inclination sensor based on a patch antenna with a reconfigurable water load. Only the water directly on the antenna is considered effective. The different volume of the effective water load, which is determined by the inclination of the attached surface, will affect the effective permittivity of the dielectric plate of the patch antenna, further causing a variation in the resonant frequency. Therefore, the proposed antenna sensor can monitor the inclination of the attached surface by interrogating the resonant frequency. The working mechanism is first clarified by theoretically investigating the relationship between the dielectric properties and the inclination of the covering medium. The antenna sensor is then simulated using High-Frequency Structure Simulator ver.15 (HFSS 15), which helps to determine geometric parameters and confirm accuracy and sensitivity. An experiment has been conducted based on the design verified in the simulation. The inclination detection shows a correlation coefficient of 0.9771 with a sensitivity of 7.92 MHz/°, indicating a potential for real application.

## 1. Introduction

Historic buildings are important all over the world and have considerable meaning in the aspects of native history and art [1,2]. To perpetuate their meanings, the preservation of their physical structures and materials stands as the most fundamental and crucial task. Nevertheless, after long-term service, historic buildings are inevitably damaged to varying degrees due to both natural and man-made factors [3,4]. The damage may gradually worsen over time and ultimately lead to irreparable destruction of historic buildings. Therefore, it is essential to continuously monitor and evaluate damage to ensure appropriate preservation and maintenance strategies of historic buildings are implemented in a timely manner. This contributes to extending the lifespan of historic buildings and enhancing their resilience to natural disasters such as earthquakes [5]. According to a standard [6], the damage level of historic buildings can be roughly judged by the inclination of the structure. To evaluate the damage level automatically, inclination detection is significant, according to the management of historic buildings [7,8,9].

Inclination detection plays an important role in many aspects of structures. (1). Construction: it can reflect surface deformation and structural changes during temporary support, excavation, filling, and foundation construction, which help to prevent accidents during construction, ensure the smooth progress of the project, and reduce risks and costs [10,11,12]. (2). Daily maintenance: inclination detection can be utilized to regularly inspect and maintain buildings or other infrastructure, contributing to more precise maintenance plans, further extending their service life [13,14]. (3). Disaster forecasting: existing structures are often threatened by natural disasters such as landslides, earthquakes, and mudslides. Continuous real-time detection of inclination can obtain the abnormal condition of the risk area, providing an important guarantee for the safety of residents and assets [15,16].

It is evident that these practical roles of inclination detection can also help to minimize the potential damage to historic buildings caused by neighboring construction, daily use, and natural disasters. On the other hand, the specificity of historic buildings and their preservation often imposes additional requirements on inclination detection. It should minimize damage on the original materials and the overall aesthetic of the historic building to avoid affecting its historic and artistic meanings.

Recently, researchers have developed various devices for inclination detection. Considering factors addressed in engineering, we summarized the characteristics of popular inclinometers as shown in Table 1. Normal mechanical inclinometers detect inclination by visual evaluation of the level of the liquid surface inside the sensor, which means there is no need to use a cable for data and power transmission. However, the data cannot be obtained remotely, which limits utilization [17,18]. With a cable for the transmission of power and data, the Fiber tilt sensor [19,20] and MEMS gyroscopes [21,22,23] enable remote sensing. Nevertheless, the cables are cumbersome for real applications. For practical utilization in historic buildings, cables may significantly disrupt the overall aesthetics, affecting their artistic value. By adding the battery inside traditional cabled sensors, several active inclinometers have been proposed. However, a much higher price and potential damage to the battery become another problem. A capable sensor for inclination detection without a cable and battery is required.

Two problems should be addressed corresponding to these requirements: (1) no batteries (passive); (2) no wire (wireless). While the traditional type of active sensor has no solutions, a patch antenna shows an approach to get rid of the above problems. This paper is focused on verification of the passive design. Acting as communication units, patch antennas can perceive changes in environmental factors through their physical state, such as deformation of relative displacement inside the antenna members. With a possible approach for passive wireless interrogation, the patch antenna has been a focal point, according to research on sensors. Currently, patch antennas have been utilized for monitoring important parameters like temperature [24], humidity [25], bolt loosening [26,27], and cracks [28,29]. They are expected to show the same potential in the detection of inclination. Nevertheless, the solid material and low profile of the patch antenna make it a complex task to convert inclination to the antenna’s physical state.

Compared with the traditional patch antenna, liquid antennas show excellent performance in tracking structural planar changes, while their usually low radiation efficiency and huge environmental effects (temperature, humidity, etc.) limit utilization in sensing [30]. Hence, rather than using liquid as the radiation member, taking the liquid as a load of the normal patch antenna, the new system can achieve plane tracking with a better performance in signal transmission [25], which is expected to enable the passive wireless detection of inclination. Nevertheless, when applying the patch antenna with the liquid load to the detection of inclination, the design and performance should be carefully analyzed. The influence of temperature is also another big concern due to the changeable dielectric ability of water. Unfortunately, there has been no research about that ever before.

This paper proposes an inclination sensor based on a rectangular patch antenna with a liquid load varied by the inclination of the attached surface. Temperature is proven to have limited influence. The resonant frequency has been proven to be sensitive to the changeable effective height of the liquid load by both simulation and experimentation. By interrogating the resonant frequency of the patch antenna, it successfully reflects changes in the liquid level, thus inverting the surface incline. This research enables the passive detection of surface inclination for the first time, laying a research foundation for future studies in intelligent construction. The potential applications of this sensor are addressed in two aspects with the expected improvements as follows:

(1). Long-term monitoring of the inclination of a large number of historic buildings. The improvements mainly concentrate on protection of historic and artistic meanings;

(2). Detection of the inclination of a specimen in a laboratory. Since the proposed antenna sensor has the potential to get rid of cables, it shows the advantage of simplifying complex installation during an experiment;

(3). Smart monitoring for normal buildings. The lower price of the proposed sensor can enable the large-scale use of the SHM system in normal buildings.

The antenna sensor is planned to be detected by a Vector Network Analyzer (VNA), which is expected to give a much more accurate performance of the proposed sensor. Hence, it should be noted that the proposed sensor can now only be verified with the ability of passive detection. The ability of wireless detection is expected to be evaluated in the future.

This paper is arranged as follows: Section 2 introduces the basic mechanism of the patch antenna. The relationship between the inclination of the covering medium and the resonance frequency is analyzed. In Section 3, an inclination sensor is designed based on the patch antenna’s mechanism. The basic parameters are determined through simulation using High-Frequency Structure Simulator ver.15 (HFSS15). The feasibility of sensors is basically proved here. The temperature effect is evaluated and proved to be limited. In Section 4, the proposed antenna sensor is fabricated. The results confirm the linear relationship between the inclination and resonant frequency, which indicate a good performance of the proposed antenna sensor. A conclusion is drawn in Section 5.

## 2. Detection Mechanism

### 2.1. Resonant Frequency of the Patch Antenna

**Figure 1:**
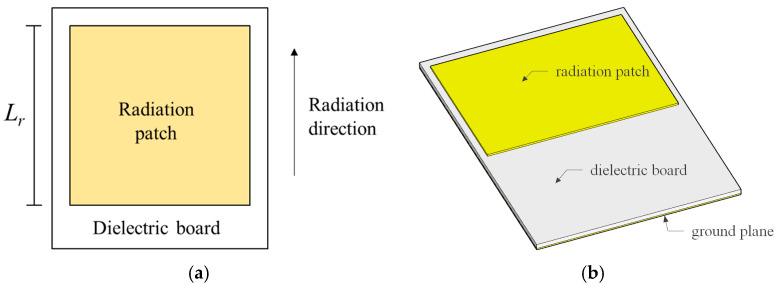
A normal rectangular patch antenna. (**a**,**b**) Top and 3D views of the patch antenna.

A patch antenna is a fundamental component in radio frequency (RF) communication systems and sensor applications. Based on the detection of deviations in their resonant frequency, a patch antenna can be utilized to perceive environmental changes. As shown in Figure 1, considering a normal patch antenna formed by a radiation patch, dielectric board, and ground plane, the resonant frequency can be calculated by Equation (1) [31]:

(1) $$f_{1}=\frac{c}{2L_{r}\sqrt{\varepsilon_{e}}}$$

where *c* is the speed of light; *L_r_* is the length of the patch antenna’s radiation patch; and *ε_e_* represents the effective dielectric constant of the substrate material. It should be noted that *ε_e_* includes the influence of the surrounding environment, which differs from the inherent dielectric constant of the antenna substrate itself. The radiation direction corresponds to the direction of the electric current of the resonance state, which is the length direction under the condition of Figure 1.

### 2.2. Influence of the Covering Material

The covering material of a patch antenna influences the effective dielectric constant of the dielectric board according to the fringe effect, further changing the resonant frequency. Based on conformal mapping, the influence can be qualitatively described as in Equation (2) [32].

(2) $$\varepsilon_{e}=g(\varepsilon_{1},h_{1})$$

where *ε*_1_ and *h*_1_ represent the dielectric constant and thickness of the covering material.

**Figure 2:**
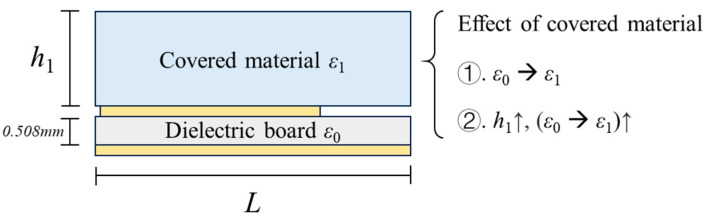
Influence of the covering material.

As shown in Figure 2, the fringe effect of the covering material can be summarized as follows:

(1) The effect tends to decrease the difference between the dielectric constant of the covering material *ε*_1_ and the dielectric constant of the dielectric board *ε*_0_.

(2) With an increase in thickness *h*_1_, the degree of the effect (increased from *ε*_0_ to *ε*_1_ as mentioned in point 1), of the covering material tends to increase. When the dielectric constant of the covering material *ε*_1_ is constant, the dielectric constant of the dielectric board *ε*_0_ is proved to vary linearly within a limited range according to Ref. [33]. The detailed relationship is described in Appendix A.

### 2.3. Design of the Inclination Sensor

The dielectric constant of the patch antenna is related to the height *h*_1_ of the covering material. When attaching the antenna to a surface, if the height of the covering material can inflect the inclination, the resonant frequency can be utilized to detect the inclination of the surface.

**Figure 3:**
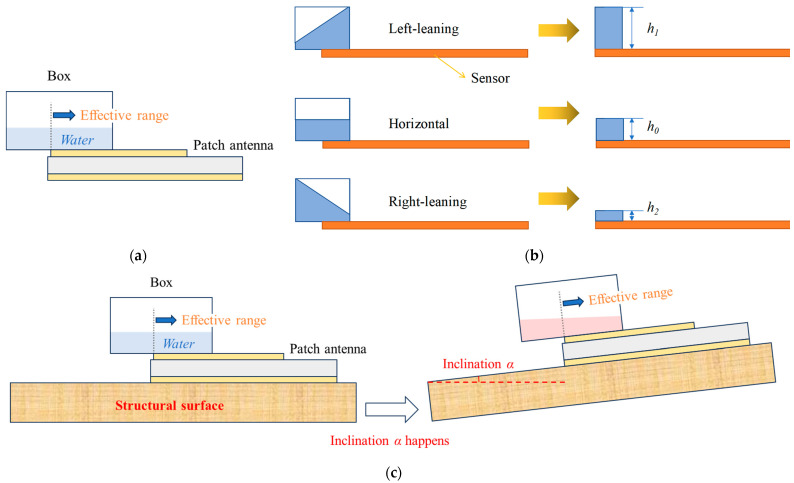
Theoretical model of the proposed inclination sensor: (**a**) basic concept; (**b**) equivalent model; (**c**) concept of the setup of the proposed sensor.

Liquid can always keep the same inclination as the attached surface. Using water as the covering material, a theoretical model of the inclination sensor designed in this paper is illustrated in Figure 3. A box with water inside is placed at the edge of the patch antenna. The water right above the patch antenna has more influence on the resonant frequency than extra water; hence, this is regarded as the effective range.

When the attached surface tilts, the antenna sensor also tilts, leading to changes in the inclination and height of the water inside the box. The inclination variations of the sensor can be divided into three stages: left-leaning, horizontal, and right-leaning states. The corresponding tilt states of covering water are depicted in Figure 3b.

The initial height of the covering dielectric is known as *h_0_.* At a horizontal state, the equivalent height of the covering dielectric can be denoted as *h_0_*. When the sensor tilts to the left, the inclination of the covering water aligns with the structural inclination, leading to a change in the water height at the effective range, denoted as *h*_1_, where *h*_1_ > *h*_0_. Similarly, when the sensor tilts to the right, the equivalent height of the covering water is *h*_2_, with *h*_0_ > *h*_2_. Since the volume of water is constant, each inclination corresponds to a specific height, which can be determined based on the equivalent volume calculation. Obviously, within a limited range of inclination, the equivalent height of covering water varied linearly with the change in inclination. The final installation is shown in Figure 3c. With the change in inclination of the attached surface, the relative angle between the surface of the water inside the box and the patch antenna is changed, which further shifts the resonant frequency.

Hence, the influence of the inclination of the covering water can be described as the following two steps:

(1). A change in inclination linearly varies the equivalent height of the covering material;

(2). A linear change in the equivalent height of the covering material varies the resonant frequency linearly (for more detail, please see Section 2.2 and Appendix A).

**Figure 4:**
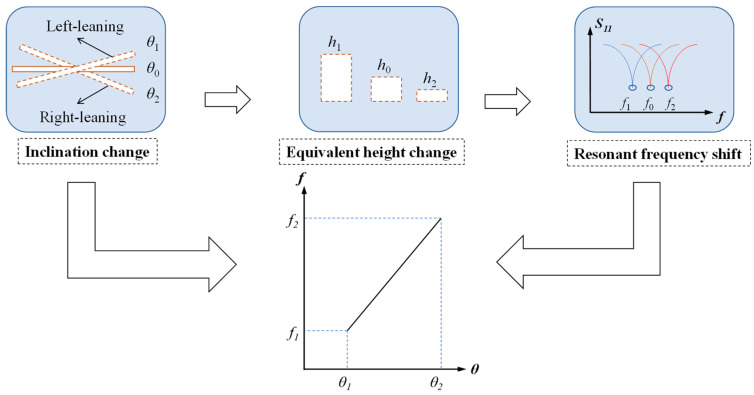
Basic mechanism of the proposed antenna sensor.

The procedure is also shown in Figure 4, which indicates that the proposed antenna sensor can detect the inclination of the attached surface using resonant frequency as the sensing parameter. However, this is only a quantitative judgment of the relationship, which should be verified further by simulation and experimentation.

## 3. Simulation

To verify the feasibility of the proposed design of the patch antenna inclination, the relationship between the inclination and the resonant frequency was calculated quantitatively by a simulation established in High-Frequency Structure Simulator ver 15 (HFSS ver 15). The setup and the results are discussed as follows.

### 3.1. Establish the Numerical Model

**Figure 5:**
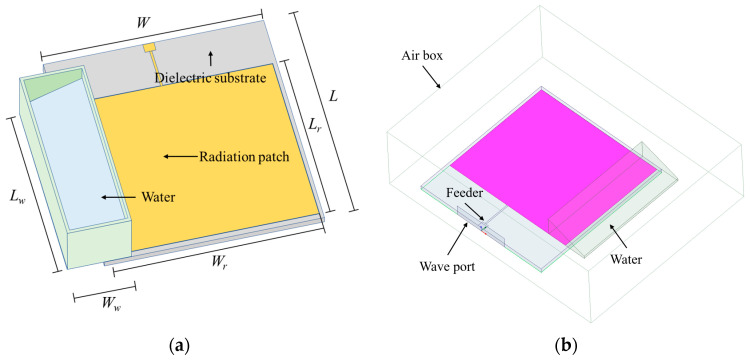
Schematic diagram of the patch antenna sensor: (**a**) basic concept; (**b**) model in HFSS.

The schematic diagram of the proposed sensor is shown in Figure 5. The model consists of a patch antenna and a covering box. The patch antenna consists of a copper sheet and a coated dielectric board. The material of the dielectric board and covering box are Rogers RT/duroid 5880 and water. The sensor is arranged in an air box with a radius of about a quarter wavelength to ensure the calculation accuracy of far-field radiation. The entire system is fed by wave port at the end of the patch antenna. Because the electric field should be completely perpendicular to the surface, perfect E was chosen as the boundary condition for the ground layer.

The geometric parameters of the patch antenna sensor are described as follows:

Patch size: the length (*L*) and width (*W*) of a patch antenna are key parameters that determine its resonant frequency, and these sizes are usually in the millimeter range.

Substrate thickness (*h*): The thickness of the medium substrate is indicated by *h*. It will affect the impedance matching and radiation characteristics of the antenna. In this paper, the substrate thickness was set at 1.6 mm.

Covering medium thickness: The thickness of the covering water is a variable parameter in the sensor design. Under the premise of a strong correlation between resonant frequency and inclination angle, the larger the height, the larger the inclination measurement range of the sensor. Nevertheless, when the height increases, the size of the patch antenna sensor is reduced. In this study, the thickness of the covering water was set to 4 mm when it is not inclinational.

The material characteristics of the proposed antenna sensor are described as follows:

Covering dielectric material: The dielectric constant of water is determined by 81, considering the property under a temperature of 20 °C [34]. The temperature effect is not discussed in this paper. The dielectric constant of the dielectric board (Rogers RT/duroid 5880) was 2.2.

After calculating the fundamental resonant frequency within a setting range by HFSS ver 15, one set of basic parameters with the best performance was obtained and is shown in Table 2.

### 3.2. Results and Discussion

#### 3.2.1. Current Distribution

**Figure 6:**
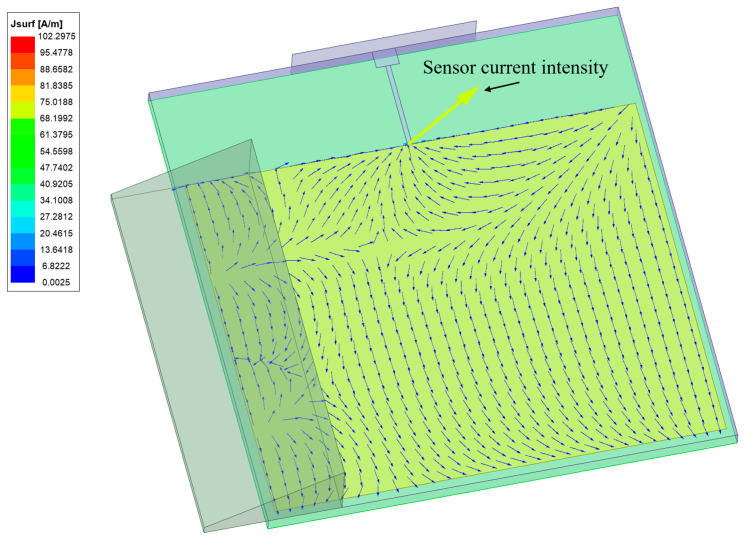
Influence of the covering material.

The induced current distribution on the patch antenna sensor at the fundamental resonant frequency is shown in Figure 6. Induced current is generated and mutates near the covering water.

#### 3.2.2. Resonant Frequency vs. Inclination

With the parameters in Table 2, the proposed patch antenna sensor was calculated. In the initial state, the liquid level of the covering medium is horizontal, indicating that the current inclination is 0 degrees. With an increase in inclinations of the sensor, the liquid surface of the covering water forms an angle with the sensor surface. The angle is up to 20°, considering the real condition of a structure.

**Figure 7:**
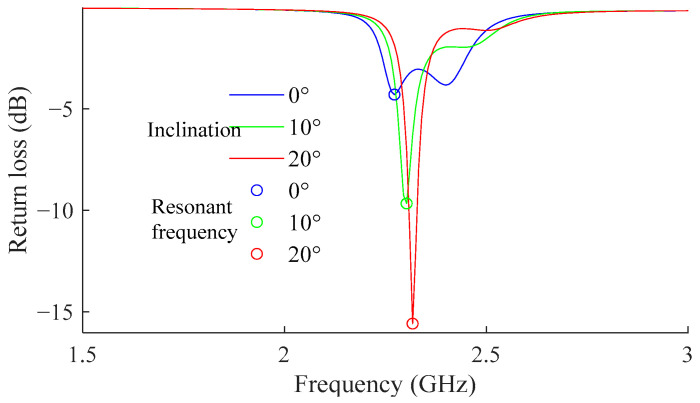
Influence of the covering material.

**Figure 8:**
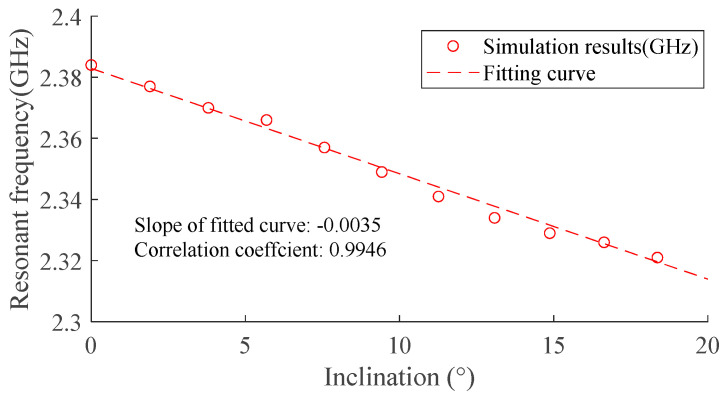
Influence of the covering material.

The step of changing the inclination was set at 2 degrees for this simulation. For each moving step, a return loss curve was obtained as shown in Figure 7. The fundamental resonant frequency of the antenna sensor under each step was then extracted from the point with a minimum return loss. The relationship between the inclination of the surface and the resonant frequency of the antenna sensor is shown in Figure 8.

As depicted in Figure 8, there is an approximate linear relationship between the inclination and resonant frequency. The correlation coefficient for inclination with resonant frequency is 0.9946, and the slope of the fitting line represents that the sensitivity of the inclination sensors is 5 MHz/°, which indicates that the proposed patch antenna sensor is capable of the detection of inclination.

Nevertheless, at the radiation boundary, the interrogation methods and environmental conditions in the simulation are slightly different from the actual situation. Hence, experiments were carried out as described in Section 4 to further verify the performance of the proposed antenna sensor.

#### 3.2.3. Temperature Influence

**Figure 9:**
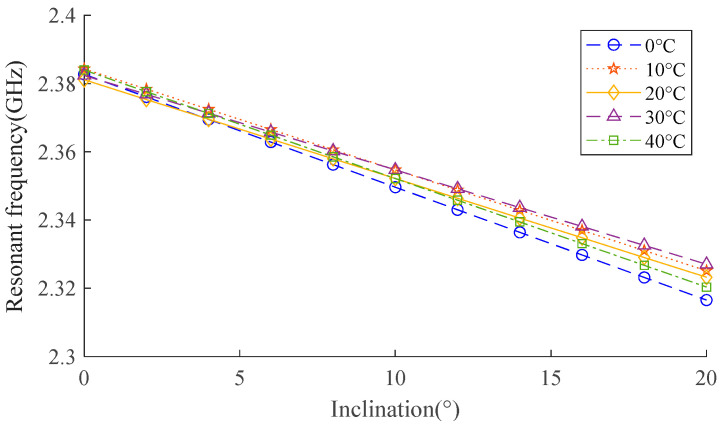
Relationship between resonant frequency and inclination of attached surface in 0–40 °C.

The dielectric constant of water is changeable, corresponding to different temperatures [34]. Hence, verifying the influence of temperature becomes important. The impact of varying temperatures on the performance of the sensor is studied in this part. Based on the setting in Table 3, the dielectric constant of the overlying liquid varies, and the relationship between temperature and the resonant frequency of the antenna sensor is displayed in Figure 9.

When the temperature is constant, the sensor’s resonant frequency maintains a linear relationship with the inclination. When a variation of 40 °C happens in temperature, there is a difference of 0.004 GHz in the initial state (inclination = 0) and 0.011 GHz when inclination increases to 20°, causing a maximum error of 2° for the observed inclination, which is still limited considering the observed range (0–20°). In the future, the model is planned to be optimized, and the way to calibrate the temperature effect is planned to be studied.

## 4. Experiments

### 4.1. Setup of the Experiment

The patch antenna was manufactured in the laboratory using a toner transfer method using a thermal printer and corrosive liquid as mentioned in Ref. [29]. Copper was chosen as the material for the radiation patch and ground plane, and Rogers RT/duroid 5880 was selected as the dielectric board of the patch antenna.

**Figure 10:**
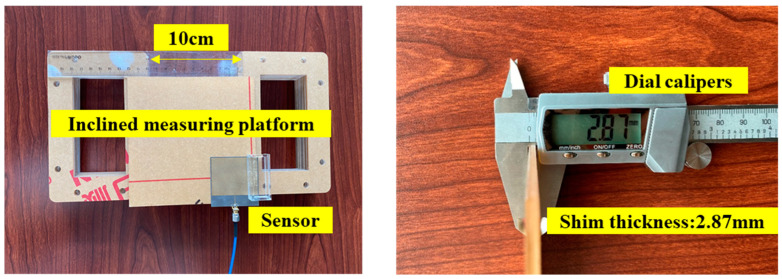
Basic parameters of the inclined measuring platform.

The fabricated antenna sensor consists of a surface, patch antenna, and small water tank. The water tank was connected to the patch antenna by glue. The basic parameters of the antenna sensor are shown in Figure 10.

**Figure 11:**
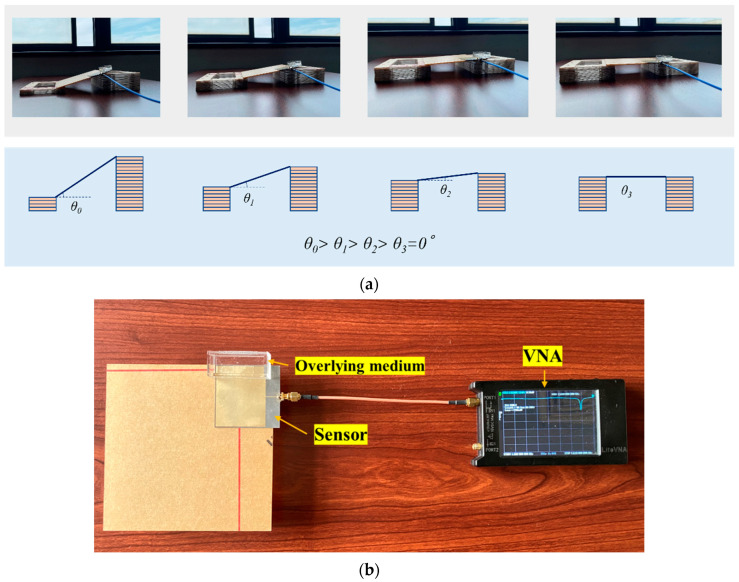
Setup of the experiment. (**a**) Concept of the device for the setup of inclination. (**b**) Top view.

An inclination simulator has been established as shown in Figure 11a. Two columns formed by several sheets were set under the patch antenna. In the initial state, the two columns were of the same height, and the surface was regarded as horizontal. By adjusting the amount of the sheet, the relative height of the column was changed, causing an inclination in the surface compared with the initial state. In this experiment, the minimum step of change in inclination was around 1° due to the parameters of the antenna sensor, attached surface, and the sheet.

A Nano Vector Network Analyzer (Nano VNA) was then utilized to analyze the return loss from the antenna sensor as clarified in Figure 11. It should be mentioned that the nano VNA decreases the measuring accuracy but increases the portability of interrogation [26], which was the reason for choosing it. The scanning range was selected from 1 GHz to 3 GHz to match the inclination sensor. Experiments were performed over an angle range of 0° to 20° to fit with the setting of the simulation. The variation of resonant frequencies was analyzed by programmatically processing the return loss of the antenna sensor at different angles. The offset of the resonant frequencies was obtained by extracting the resonant frequency at the local minimum of each S11 curve, which is described in Section 4.2.

### 4.2. Analysis and Interpretation of the Experimental Result

**Figure 12:**
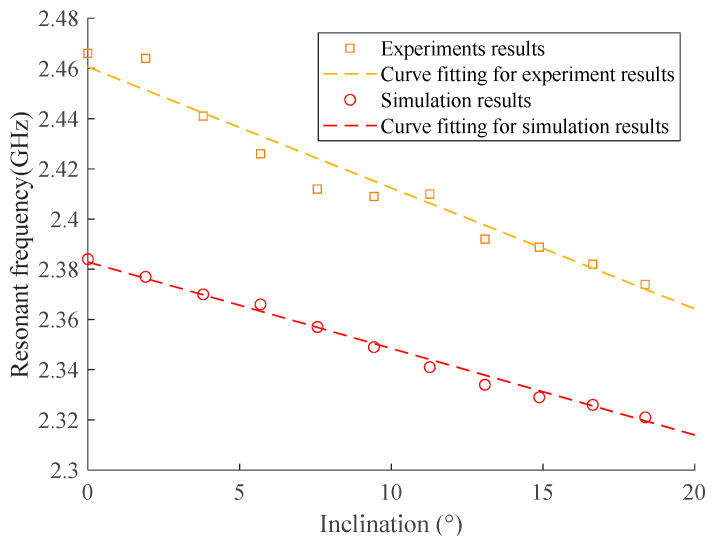
Numerical simulations and experiments of resonant frequency versus inclination angle.

Figure 12 shows the relationship between the fundamental resonant frequency and the angle, both for the numerical simulation and experiment, and the results of sensitivity, measurement ranges, and correlation coefficients (*r*^2^) of fitted lines for the two cases are compared in Table 4. Although the initial fundamental resonant frequencies are slightly different from each other due to the difference between the experiment and simulation, the sensitivity shows good consistency, which indicates the simulation can have a good reflection of the real behavior of the antenna sensor. The correlation coefficients of all fitting lines are above 0.95, indicating the great workability of the antenna sensor.

It should be noted that though the initial resonant frequency stays kind of the same with a difference of 3%, there was a difference of 30% between the sensitivity of the simulation and experiment results. The difference in sensitivity may be due to the air gap between the covering medium and the patch antenna. In addition, each method has a slightly different measurement range. This difference may be due to the following reasons:

(1) The influence of the inclination simulator itself is ignored in numerical simulation and theoretical calculation. However, the influence of the inclination simulator itself is considered in the experiments.

(2) The use of micrometers produces electromagnetic interference, which is not considered in theoretical calculations or numerical simulations.

The difference between the initial resonant frequency and the sensitivity indicates the importance of initial calibration, which is planned to be discussed as one part of the next step.

## 5. Conclusions

Inclination is essential for the long-term health monitoring of historic buildings. Nevertheless, traditional wired sensors cannot ensure aesthetics and further decrease historic and artistic meanings. This paper presents a novel inclination sensor based on a patch antenna. Based on the fringe effect and the fluid characteristic of covered water, the resonant frequency of the proposed patch antenna is proved to be related to the angle of the attached surface, which indicates the sensor is capable of detecting inclination. The resonant frequency is selected as the sensing parameter for the detection of inclination, which is easy to achieve via passive wireless interrogation.

Both theoretical analysis, numerical simulation, and an experiment were carried out to prove the feasibility of the proposed antenna sensor. Two main conclusions can be obtained based on the analysis of this paper:

(1) Both simulation and experimentation show a linear relationship between the inclination of the attached surface and resonant frequency of the patch antenna, with a correlation coefficient higher than 0.95, which indicates that the proposed patch antenna sensor is capable of detecting inclination.

(2) The proposed patch antenna sensor has a good consistency of initial resonant frequency among the simulation and experiments with a difference of 3%, indicating that the numerical model is accurate for further development of the patch antenna. However, the sensitivity between the simulation and experiment has a difference of 30%, which is not bad but requires research for calibration.

The future target is summarized as follows:

(1). Wireless interrogation of the proposed patch antenna sensor is planned to be investigated to improve the ability for practical utilization.

(2). In order to improve accuracy, the calibration toward temperature and other possible effects is planned to be carried out.

## Figures and Tables

**Table 1:** Comparison of detection methods.

Type	Accuracy	Power	Cable	Cost ($)	Ref.
Mechanical inclinometer	△	×	×	2~20	[17,18]
MEMS gyroscopes	○	√	√	/	[19,20]
Fiber tilt sensor	○	√	√	/	[21,22,23]

Influence: ○ > △; required (√); not required (×).

**Table 2:** Parameters of the inclination sensor.

Parameters	*W*	*W_w_*	*W_r_*	*L*	*L_w_*	*L_r_*
Dimensions (mm)	51	15	49	54.3	42.3	41.3

**Table 3:** The relationship between temperature and the dielectric constant of water.

Temperature	0 °C	10 °C	20 °C	30 °C	40 °C
Dielectric constant	87.740	83.832	80.103	76.546	73.151

**Table 4:** Sensitivity, measuring range, and *r*^2^ of the fitted line: numerical simulation and experiment.

Serial	Sensitivity (MHz/°)	Initial Resonant Frequency (GHz)	Measuring Range (GHz)	Correlation Coefficient (*r*^2^) of the Fitted Line
Numerical simulation	5.5 MHz	2.467	2.384–2.318	0.9946
Experiment	7.92 MHz	2.385	2.466–2.371	0.9771
Difference	30%	3.4%	/	/

**Figure A1:**
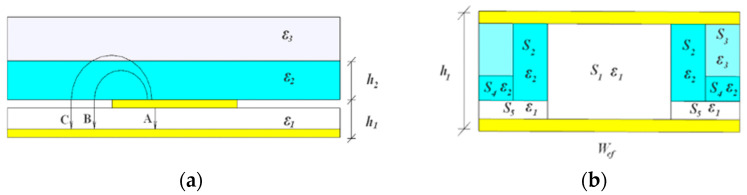
Equivalent model using electric flux theory. (**a**) Electric flux path and (**b**) equivalent patch antenna.

## Data Availability

The data are available based on requirement.

## Appendix A

Appendix A describes the calculation method for the resonant frequency of a patch antenna with a covered material, which is related to a reference [33]. Consider the model in Figure A1.

First, the antenna width will be enlarged due to the nonlinear flux path. The effective width *W_ef_* of the equivalent patch antenna can be estimated as follows:

(A1) $$W_{ef}=W+(2\times h_{1}/\pi )\times \ln[17.08(w/2\times h_{1}+0.92)].$$

Then, the filling fractions *q*_1_, *q*_2_, and *q*_3_, representing the capacitive effect of the three regions with different dielectric constants, are expressed as follows:

(A2) $$q_{1}=1-\frac{h_{1}}{2W_{ef}}\ln(\frac{\pi }{h_{1}}\times W_{ef}-1),$$

(A3) $$q_{2}=1-q_{1}-\frac{h_{1}-v_{\varepsilon }}{2W_{ef}}\ln[(\frac{\pi }{h_{1}}\times W_{ef}\frac{\cos(\frac{\pi v_{\varepsilon }}{2h_{1}})}{\pi (\frac{h_{2}}{h_{1}}-\frac{1}{2})+\frac{\pi v_{\varepsilon }}{2h_{1}}}+\sin(\frac{\pi v_{\varepsilon }}{2h_{1}})],$$

(A4) $$q_{3}=\frac{h_{1}-v_{\varepsilon }}{2W_{ef}}\times \ln[(\frac{\pi }{h_{1}}\times W_{ef}\frac{\cos(\frac{\pi v_{\varepsilon }}{2h_{1}})}{\pi (\frac{h_{2}}{h_{1}}-\frac{1}{2})+\frac{\pi v_{\varepsilon }}{2h_{1}}}+\sin(\frac{\pi v_{\varepsilon }}{2h_{1}})],$$

where the quantity *v_ɛ_* is given as follows:

(A5) $$v_{\varepsilon }=\frac{2h_{1}}{\pi }\arctan[\frac{\pi }{\frac{\pi \times W_{ef}}{2h_{1}}-2}(\frac{h_{2}}{h_{1}}-1)].$$

Due to ignorance of the filling fraction expressions in the ultimate limit state when there is no covering material, Equations (A1)–(A5) overestimate the effect of the second layer dielectric board with a dielectric constant of *ɛ*_2_. To rectify the error, a new filling fraction *q*_4_ is proposed by Equation (A6).

(A6) $$q_{4}=(h_{1}/2W_{ef})\times \ln(\pi /2+h_{1}/2W_{ef}).$$

Then, two filling fractions, $q_{1}$ and $q_{2}$, are modified as $q_{1n}$ and $q_{2n}$ as follows:

(A7) $$q_{1n}=q_{1}-q_{4}.$$

(A8) $$q_{2n}=1-q_{1n}-q_{3}-2q_{4}.$$

The effective dielectric constant *ɛ_e_* of the covered antenna can be calculated as follows:

(A9) $$\varepsilon_{e}=\varepsilon_{1}q_{1n}+\frac{\varepsilon_{1}{\left(1-q_{1n}\right)}^{2}\times [\varepsilon_{2}^{2}q_{2n}q_{3}+\varepsilon_{2}\varepsilon_{3}(q_{2n}q_{4}+{\left(q_{3}+q_{4}\right)}^{2})]}{\varepsilon_{2}^{2}q_{2n}q_{3}q_{4}+\varepsilon_{1}(\varepsilon_{2}q_{3}+\varepsilon_{3}q_{4}){\left(1-q_{1n}-q_{4}\right)}^{2}+\varepsilon_{2}\varepsilon_{3}q_{4}[q_{2n}q_{4}+{\left(q_{3}+q_{4}\right)}^{2}]}.$$

Considering the effect of effective length extension, the adjusted effective dielectric constant $\varepsilon_{e}^{\prime }$ is rewritten as follows:

(A10) $$\varepsilon_{e}^{\prime }=(2\varepsilon_{e}-1+K)/(1+K),$$

where *K* is as follows:

(A11) $$K=\sqrt{W_{ef}/(W_{ef}+10h_{1})}.$$

Finally, the resonant frequency *f* of the equivalent patch antenna is obtained as follows:

(A12) $$f=cn/(2L_{r}\sqrt{\varepsilon_{e}^{\prime }})$$

where *c* is the speed of light, and *n* is the order of resonant frequency.
